# Involvement of immune responses in pulmonary arterial hypertension; lessons from rodent models

**DOI:** 10.1186/s42826-019-0021-1

**Published:** 2019-11-06

**Authors:** Kibyeong Kim, Jae-Hoon Choi

**Affiliations:** Department of Life Science, College of Natural Sciences, Research Institute of Natural Sciences Hanyang University, Seoul, Republic of Korea

**Keywords:** Pulmonary hypertension, Immune cells, Inflammation, Rodent models, Pulmonary arterial hypertension

## Abstract

Pulmonary hypertension (PH) is a pathological state with sustained elevation of pulmonary artery (PA) pressure. Since the pathogenesis of PH is mostly irreversible, the disease often comes up with poor prognosis. Pulmonary arterioles are affected by deteriorative changes, such as development of occlusive lesions of thickening of arterial walls. Such processes increase the pulmonary arterial pressure thus lead to consequent injuries such as right ventricle failure. Proliferation, or resistance to apoptosis of pulmonary artery smooth muscle cells (PASMC) and fibroblasts, are characteristic changes observed in the PA in pulmonary arterial hypertension (PAH) patients. PAH can either occur idiopathically or come with other diseases. Emerging evidences suggest that pro-inflammatory processes are closely related to the development of PAH. Therefore, it is inferred that immune cells could be the key factors in PAH development. In this review, we summarize the way how each types of immune cells participate in PAH. We would also like to list the current rodent models used for PAH study.

## Introduction

Pulmonary arterial hypertension (PAH) is a pathological state characterized by sustained elevation of pulmonary artery (PA) pressure; it is diagnosed when the mean resting PA pressure exceeds 25 mmHg or the mean exercise PA pressure exceeds 30 mmHg [1]. Common PAH symptoms include dry cough, vomiting, respiratory failure, fatigue, and dizziness, which are exacerbated by physical activity or exercise [2]. Occlusive changes take place in the affected pulmonary arterioles and increase the pulmonary arterial pressure [3, 4]; these changes are due to the proliferation or resistance to apoptosis of pulmonary artery smooth muscle cells (PASMC) and fibroblasts, which are characteristic changes observed in the pulmonary artery in PAH patients [4]. As the disease progresses, pulmonary resistance and right ventricular load increase, and compensatory changes take place, such as right ventricular hypertrophy, which ultimately causes right ventricle failure and results in death [5]. PAH has a poor prognosis, with 90 and 40% one- and three-year survival rates, respectively [6]. PAH patients complicated by other diseases, like systemic sclerosis, have an even worse survival rate [7].

To date, several processes including endothelial dysfunction, smooth cell and fibroblast hyperplasia, vasoconstriction, and recruitment of mesenchymal progenitor cells have been assumed to be involved in PAH; in many cases, the cause cannot be specified [4]. PAH can either be associated with other diseases, such as human immunodeficiency virus (HIV) or systemic sclerosis, or it can occur idiopathically [8, 9]. Bone morphogenic protein type II receptor (BMPR2) is the most vastly studied PAH-inducing factor and has been described as a major influence in the development of PAH [10]; more than 26% of IPAH patients have a mutation in the *BMPR2* gene [11]. PAH patients with a heterozygous *BMPR2* mutation exhibit decreased *BMPR2* expression [12]. Importantly, in a mouse model, an endothelial-specific BMPR2 deficiency was sufficient to induce PAH [13].

Notably, recent studies have revealed that inflammation is closely connected to PAH [14]. BMPR2 malfunction induces interleukin-6 (IL-6) overexpression [15], which can produce PAH [16, 17]. In addition, a decrease in BMPR2 expression leads to translation of proinflammatory cytokine GM-CSF and the infiltration of macrophages into the muscularized vessels [17]. Selective enhancement of BMPR2 by BMP9 inhibits JNK phosphorylation by TNFα in pulmonary artery endothelial cells (PAECs) and prevents PH progression of BMPR2-deficient mice, which are prone to PH [18]. In addition to the previously mentioned cytokines, leukotriene B4, a pro-inflammatory cytokine secreted mainly by macrophages, is also elevated in the macrophages and endothelial cells of IPAH patients. PH model mice also showed increased plasma LTB4 level [19] with PAEC apoptosis, which is a trigger of PH. Moreover, administration of an LTB4-synthesizing enzyme inhibitor ameliorated PH lesions [20]. Lung-specific overexpression of IL-6 caused right ventricular hypertrophy (RVH) and an increase in the right ventricular systolic pressure (RVSP), along with pulmonary arteriole muscularization that was associated with increased vessel wall thickness or complete occlusion. Histological analysis revealed that T cell recruitment to the perivascular areas contributed to vessel occlusion [16]. In contrast, IL-6 knockout mice showed improved hypoxia-induced RVH and an increase in RVSP with decreased pulmonary arteriole wall thickness [21]. Collectively, pro-inflammatory processes are intimately involved in the development of PAH. Therefore, immune cells are important players in PAH development. In this review, we focus on the immune cell functions in PAH. Moreover, we would like to summarize the current animal models for PAH study.

## Main text

### Macrophages

A number of studies have indicated the infiltration of macrophages near the lesion areas in and around the blood vessels in PAH [13, 20, 22–24]. Macrophages are myeloid immune cells that are involved in innate immunity via phagocytic activity and cytokine production. They reside in the peripheral tissues, including the alveoli and vessel walls of the lungs. Any stimulus that causes PAH also induces macrophage infiltration, which initiates inflammation and production of cytokines responsible for further inflammation and arteriole muscularization. Moreover, macrophage blockade via the suppression of recruitment, functioning, or production can ameliorate PAH [17, 20, 23]. Early macrophage infiltration into the affected arterioles is crucial for the development of PAH because macrophages contribute to the pathogenesis of PAH by damaging endothelial cells and by secreting factors that promote arteriole muscularization, such as VEGF and PDGF [23, 24]. Macrophages participate in PAH development in experimental PAH as well as in clinical cases; these cells infiltrate the pulmonary arterioles in IPAH patients [17].

### Dendritic cells

Dendritic cells (DCs), which are the most potent antigen-presenting cells, are also implicated in PAH. These cells engulf and process the antigens to be presented on their surfaces so that helper T cells can recognize them and initiate the adaptive immune response. Although the functions of DCs in many diseases have been determined, the role of DCs in PAH is still poorly understood; only one study has demonstrated the involvement of DCs in human PAH by showing that DC-SIGN^+^ DCs primarily accumulate in the adventitial connective tissues of PAs in IPAH patients [25]. These cells had the profiles of immature DCs. DCs were less common in the lung parenchyma and were absent in the pulmonary venules and capillaries. In PAH model rats, DCs increased in number from 14 days after monocrotaline (a toxic agent widely used to induce PAH in experimental animals) administration and a significant number of DCs was recruited to the adventitial layers of PAs from Day 28. DC number was positively correlated with lesion severity [25]. Furthermore, the accumulation of c-kit+ cells has been shown in the pulmonary vessels of PAH patients. Some of the cells expressed major histocompatibility complex class II (MHC class II) [26]. Since some DC populations can express c-kit [27], it is plausible that the c-kit+ MHC class II+ cells that accumulate in the PA lesions may be DCs. Additionally, DCs produce chemokine CX3CL1. Chemokine production increases as DCs undergo maturation [28]. CX3CL1 promotes smooth muscle cell proliferation in PAH [29], implying that DCs are somehow responsible for PAH pathogenesis and development. However, as previously stated, few studies have specifically investigated the detailed mechanisms by which DCs participate in PAH pathogenesis and development. Thus, their actions should be inferred using indirect evidence, such as the means by which T cells are involved in PAH, for DCs actively interact with T cells to regulate their activity.

### T cells

T cells are key compartments in cellular immunity that participate in the immune response by directly attacking targets, by enhancing other immune cells, or by suppressing excess immune responses. T cells are likely closely connected to PAH, although it is not clear whether T cells initiates PAH or just involve the progression of PAH. Indeed, the basis by which immune dysregulation induces PAH has received little investigation [30]. Nevertheless, there has been remarkable progress in this area. For instance, animals that lack T cells are more susceptible to severe pulmonary hypertension [31, 32], which is attenuated by immune reconstitution with spleen cells, more specifically, CD4+ T cells. Accordingly, an anti-CD4 monoclonal antibody, which significantly reduces the number of CD4+ T cells, also worsened PAH development. The introduction of CD8+ T cells was found to have no beneficial effect. CD4+ T cells with protective roles were identified as regulatory T cells (Tregs) because they migrate to the lungs in the early stages of PAH development to suppress pulmonary inflammation. They also enhanced lung BMPR2 expression and suppressed endothelial cell apoptosis, supporting the notion that Tregs have protective effects on the development of PAH [33]. Clinically, it has been reported that the number of Tregs is decreased in IPAH patients; PA endothelial cells secrete leptin, which suppresses Treg activity [34]. Previously, DCs have been found to be highly responsible for Treg generation. Therefore, the protective activity of Tregs on PH may be controlled by DC populations in the lung during the development of PH, which remains to be determined in future studies.

In addition to Tregs, Th2 cells are also related to PAH. Though they did not actually increase RVSP, the Th2 immune response caused muscularization of the pulmonary arterioles via resistin-like molecule (RELM) in an α-dependent way [35]. The nuclear factor of activated T cells (NFAT), a key activating factor of T cells, is also described in PAH. T cells were detected in the remodeled PAs of PAH patients, and most T cells demonstrated NFATc2 activation. Furthermore, such activation was also found in the blood, small PAs, and PASMCs in human PAH patients. A protective effect was also observed when NFAT was indirectly inhibited by cyclosporine A, indicating that T cells and their activating factors are correlated with the pathogenesis of PAH [36].

### B cells

B cells participate in adaptive immunity by producing several types of immunoglobulins, including the secreted form IgG, which is commonly known as an antibody. There is little evidence that B cells are directly involved in PAH. However, B cells are implied in PAH in the context of autoimmunity [30]. Patients with anti-endothelial cell antibodies (AECA) are at significantly higher risk of PAH [37]. Tregs, which are crucial in tolerance and in regulation of excessive immune responses, showed a clear effect in downregulating in vitro B cell proliferation induced by lipopolysaccharides and on inhibiting the production of autoantibodies [38]. Athymic rats, which lack T cells (including Tregs), showed increased inflammation in B cells; immune reconstitution with Tregs also attenuated the disease [33]. Therefore, one of the mechanisms suggests that Tregs prevent PAH and should be connected to Treg-B cell interactions.

### Natural killer cells

Natural killer (NK) cells are commonly considered to participate in innate immunity by specifically attacking cells infected by viruses or cells being transformed into cancer cells without the use of antigen recognition. Interestingly, many studies have proposed that NK cells are involved in vascular remodeling and regeneration [39–41]. Moreover, the finding that NK cells play a significant role in the pathogenesis of HIV supports the notion that NK cells are associated with the pathology of PAH, as HIV infection is occasionally accompanied by PAH [8, 41, 42]. Motivated by these facts, a group of researchers investigated how NK cells are associated with PAH and found that fully cytotoxic CD56^dim^/CD16+ NK cells decreased in number, and NK cell function in PAH patients was impaired. Accordingly, NK cell number and function also decreased in experimental rat PAH models [41].

### Neutrophils

Neutrophils circulate through the bloodstream and engulf the antigens they encounter and comprise a key component of innate immunity. However, little is known about how neutrophils contribute to the progression of PAH. A previous study demonstrated that neutrophil elastase (NE) is produced by pulmonary artery smooth muscle cells and is linked to neointimal lesions [43]. NE was originally considered to be an antibacterial protein necessary for eliminating Gram-negative bacteria [44]. In that study, PA elastin and the elastic laminae of S100A4-overexpressing mice were observed to be prone to disintegration due to an increase in serine elastase activity. NE had an equivalent physiological role to serine vascular elastase, and the administration of NE-inhibiting elafin prevented PA neointimal lesion development. NE expression was also significantly increased in IPAH patients [43].

### Rodent models of pulmonary hypertension

It is essential to establish a robust animal model for a biological research, and that is also true for PH. To date, several animal models for pulmonary hypertension have been established. Among those, chronic hypoxia model and monocrotaline injury model are most commonly used animal models that contributed fairly in revealing the pathophysiology of pulmonary hypertension [45]. Monocrotaline injury model was first developed in 1967 [46] and since then it is widely applied in PAH research. Monocrotaline is a pyrrolizidine alkaloid with toxicity. It is usually found in plant *Crotalaria spectabilis*. It is reported that in many animal species, administration of monocrotaline causes progression of PH. Monocrotaline has to be metabolized to monocrotaline pyrrole (MCTP) by a combination of oxidases in order to have activity of inducing the disease. The exact mechanism by which MCTP induces PH is yet to be discovered. Nevertheless, it is well reported that MCTP could damage the vascular endothelia, and in turn, it is inferred that the affected endothelial cells provoke inflammation along with surrounding vascular smooth muscle cell proliferation to develop a PH-characteristic obliterative vascular lesions. Monocrotaline rat model has an advantage in representing severe pulmonary vascular lesions and right ventricle hypertrophy that are very similar to those observed in human patients.

In contrast, the attempts for establishing mice model with monocrotaline were found not successful, that monocrotaline has to be metabolized into its active form dehydromonocrotaline for disease induction [47], and that process requires expression of Cyp3a isoenzyme, which is functionally affected in mice. Hence, chronic hypoxia model is more commonly used in mouse for pulmonary hypertension studies [45, 48]. Hypoxic mice model showed rather lower disease severity than that of rats [49]. To cope with this limitations, early mice PH studies focused on genetically modified mice. One study exposed 5-lipoxigenase (5-LO) knockout mice to hypoxia, and the authors found that such weak disease induction in mice than rats, along with the observation that 5-LO knockout mice showed further relieve in disease development [49]. Another study [50] investigated whether impairment in *Bmpr2* gene expression in smooth muscle cells itself could affect the pathogenesis of PH, since previous clinical studies suggested that a large proportion of familial/idiopathic PAH patients carried mutations in *BMPR2* gene, thus such mutation could be an important PH-inducing factor. Smooth muscle cell-specific *Bmpr2* knockout mice exhibited mild increase in RVSP along with muscularization in medial layer. However, severe intimal cell proliferation, a distinctive feature of PAH patients’ lungs, was not detected [50]. In 2008, Hong et al. worked on mice with pulmonary vascular endothelial cell-specific loss of *Bmpr2* [13]. The authors chose such model since systemic *Bmpr2* knockout is embryonically lethal. Conditional knock-out process involved mating with *Alk1*-Cre mice with *Bmpr2*^*fl/fl*^ mice. Endothelial cell-specific deletion of *Bmpr2* caused spontaneous PH with vascular remodeling in some individuals [13]. However, RVSP varied largely (20–56 mmHg) among individuals that only one third of the animals were certainly diseased. Moreover, the severity of RVH from individuals with apparent RVSP increase was lower than expected. In addition to the methods with genetically modified mice, there was another attempt that tried applying one of disease-inducing procedures that is originally used in rats to mice models. Along with 3 weeks of hypoxic conditions, a VEGF receptor blocker, SU 5416, is applied subcutaneously to mice [51]. Mice exposed with hypoxia plus VEGFR inhibitor showed higher right heart hypertrophy indices, right ventricle pressure and vascular muscularization factors than those of groups exposed to only either of such disease-inducing conditions. During exposure to hypoxia and SU 5416, Caspase-3-expressing endothelial cell per vessel ratio and PCNA+ endothelial cell per vessel ration significantly increased when compared to control. Pulmonary artery RNA profiling of transforming growth factor-β/bone morphogenetic protein pathway genes revealed that *Bmpr2*, a well-known PH-related gene, significantly decreased. On the other hand, transcription of TGF-β-related factors such as *Pai1*, *Pdgfr* and *Tph1*, and inflammatory genes i.e. *Il6*, *Hif1a* significantly upregulated. Whole-lung Western Blot Analyses showed increase of PSmad1/2 proteins and decrease in levels of phosphorylated Akt proteins.

## Conclusions

It is now clear that the early diagnosis of PAH significantly enhances patient survival. Despite its importance, early PAH diagnosis is difficult because the signs of the disease in its earliest stages are not apparent in many cases. Even mild elevations in pulmonary arterial pressure can reflect diffuse and extensive vascular damage. Changes in right ventricular function and structure, which can be assessed using noninvasive diagnostic methods, occur later in the clinical course of PAH [52]. However, noninvasive diagnostic methods are not as accurate as cardiac catheterization, which is quite invasive and therefore is not appropriate for baseline screening. As we discussed in this review, since many immunological processes are involved in the pathogenesis of PAH, it is needed to define PAH-specific immune cells or factors which can be monitored in serum or total blood, to make successful early PAH diagnosis. And it appears that rodent PAH models are highly valuable for understanding the pathogenesis of PAH and identify novel PAH markers for early detection of PAH.

## Data Availability

Not applicable.

## Abbreviations

5-LO: 5-lipoxigenase
AECA: Anti-endothelial cell antibodies
BMPR2: Bone morphogenic protein type II receptor
HIV: Human immunodeficiency virus
IPAH: Idiopathic pulmonary arterial hypertension
LTB4: Leukotriene B4
MCTP: Monocrotaline pyrrole
NE: Neutrophil elastase
NFAT: Nuclear factor of activated T cells
NK cell: Natural killer cell
PA: Pulmonary artery
PAEC: Pulmonary artery endothelial cell
PAH: Pulmonary arterial hypertension
PASMC: Pulmonary artery smooth muscle cell
PCNA: Proliferating cell nuclear antigen
PDGF: Platelet-derived growth factor
PH: Pulmonary hypertension
RELM: Resistin-like molecule
RVH: Right ventricular hypertrophy
RVSP: Right ventricular systolic pressure
Treg: Regulatory T cell
VEGF: Vascular endothelial growth factor
